# The synthesis of triazine–thiophene–thiophene conjugated porous polymers and their composites with carbon as anode materials in lithium-ion batteries†

**DOI:** 10.1039/d0ra10862f

**Published:** 2021-03-11

**Authors:** Xin Xue, Junming Luo, Lingqian Kong, Jinsheng Zhao, Yan Zhang, Hongmei Du, Shuang Chen, Yu Xie

**Affiliations:** State Key Laboratory of Heavy Oil Processing, College of Chemical Engineering, China University of Petroleum (East China) QingDao 266580 P. R. China chsh1030@163.com; College of Chemistry and Chemical Engineering, Liaocheng University Liaocheng 252059 P. R. China j.s.zhao@163.com; School of Environmental and Chemical Engineering, Nanchang Hangkong University Nanchang 330063 China xieyu_121@163.com; Dongchang College, Liaocheng University Liaocheng 252059 P. R. China

## Abstract

The polymers based on thiophene armed triazine and different thiophene derivatives including thiophene (Th), thieno[3,2-*b*]thiophene (TT), dithieno[3,2-*b*:2′,3′-*d*]thiophene (DTT) or thieno[2′,3':4,5]thieno[3,2-*b*]thieno[2,3-*d*]thiophene (TTTT) are synthesized through a Stille coupling reaction. By introducing thiophene derivatives with increasing sizes as the linkage units (from thiophene, DT to DTT, TTTT), the band gaps (*E*_g_) of the resultant polymers decrease continuously. Then the composite materials (polymer@C) between polymers and Vulcan XC-72 carbon are prepared by *in situ* polymerization to test their electrochemical performances in lithium ion batteries. The synthesized composites show distinct morphologies due to the different linkage units of thiophene or fused cyclothiophene derivatives and the cross-linked structure can be found in composites with the longer thiophene derivatives (bridging molecules) like PTT-3@C and PTT-4@C, which are expected to be beneficial to improve the performances of the electrode materials. The specific capacities of the composites are 495 mA h g^−1^, 671 mA h g^−1^, 707 mA h g^−1^, and 772 mA h g^−1^ for PTT-1@C, PTT-2@C, PTT-3@C and PTT-4@C at a current density of 100 mA g^−1^, respectively. In particular, benefiting from the enlarged conjugation length and planarity of the linkage units, the conjugated microporous polymers could deliver continuously improved capacities.

## Abstract 2

Four different kinds of conjugated porous polymers PTTs were synthesized and their composites with carbon material were used as the electrode materials for LIBs.

## Graphical abstract

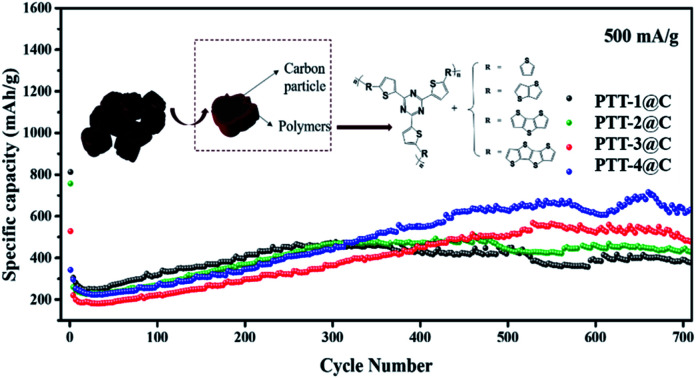

## 1. Introduction

Since Sony commercialized the first lithium ion batteries (LIBs) with carbon anodes in 1991, this novel battery has been quickly applied in many fields in our daily life for its intrinsic merits like being environment-friendly, and having high energy density and long cycle life.^1^ Nowadays, with the increasing demand for clean energy and quick-acting charging,^2^ LIBs are now facing more requirements and challenges. Despite the low-cost and stability, the conventional carbon anodes still have many limitations, among which the most unsatisfactory one is the low capacity, only 372 mA h g^−1^ in theory.^3^ Thus, the exploration towards better electrode materials for LIBs has never stopped. Besides inorganic materials, organic polymers which have variable molecular structures are also promising electrode materials for application in LIBs.^4^ And one of the most important advantages of organic polymers is their molecular engineering, which allows the structures to be adjusted or modified on the molecular level in order to achieve some desirable goals.^5^

Dissolution in the electrolyte is one of the main drawbacks of organic compounds, which can lead to the capacity fading during cycling process.^6^ Various methods have been explored to solve the dissolution problem, such as the polymerization of small molecules^7,8^ and anchoring organic compounds on the conductive carbon matrix,^9^ both of which are aimed at giving the organic materials a hard and stable backbone. Another problem is the low conductivity, organic molecules are naturally weak at electrical conductivity due to its strong intramolecular covalent interaction.^5,10^ Carbon materials like carbon nanotube (CNT),^11^ graphene,^12^ graphite^13^ or just carbon powder are all good non-metal conductors, the addition of carbon materials to organic based materials can increase the conductivity of the resultant composites. On the other hand, unlike the inorganic materials, the lack of free electrons and ions limits the transportation of charge in organics, but the polymerization of some π-conjugated aromatic compounds^6^ with lone-pair electrons can facilitate the delocalization of π electrons in the whole molecule, thereby offset the lack of conductivity, such like polythiophene (PT),^1^ polypyrrole (PP)^14^ and their derivatives, which have been proved electroactive in rechargeable batteries.

The main factor that should be considered into the molecular design of the polymers for electrode usage is the orbital energy level. According to the frontier molecular orbital theory, the reactive activity of the polymers depends on their frontier molecular orbital, that is, the HOMO (highest occupied molecular orbital)/LUMO (lowest unoccupied molecular orbital) and the band gap energy (*E*_g_), which is the crucial factor determining the electronic and conducting properties of conjugated polymers.^15^ A narrow band gap is always desired for polymers as anode materials, it can be achieved by raising HOMO energy level and lowering LUMO energy level.^16^ For this, the donor–acceptor (D–A) type polymers, which is characterized by introducing electron-rich donor (D) units and electron-deficient acceptor (A) units into the polymers at the same time, has been usually adopted for greeting low bandgap polymer.^17^ Thiophene with various substitution patterns are the commonly used electron-donating moieties. Zhang *et al.* reported the different electrochemical performances of the thiophene-containing polymers, among them, poly(3,3′-bithiophene) (P33DT) with crosslinked structure could even exhibited the specific capacity of around 1215 mA h g^−1^ at 45 mA g^−1^.^1^

Triazine and its derivatives have attracted intensive attentions for their strong electron-deficient property due to the presence of three pyridinic-N atoms in their structures. As reported by Ken Sakaushi *et al.*,^18^ the covalent triazine-based framework from *p*-dicyanobenzene (ACTF-1) generated a specific capacity of 160 mA h g^−1^ at 0.05 A g^−1^. In a report by Kang *et al.*^19^ the copolymer containing both 2,4,6-trichloro-1,3,5-triazine (TCT) and 2,6-diamino anthraquinone (AQ) was prepared, the synergistic effect between the two units was noticed and the resultant copolymer exhibited a high capacity up to 3000 mA h g^−1^ at 200 mA g^−1^.

**Fig. 1:**
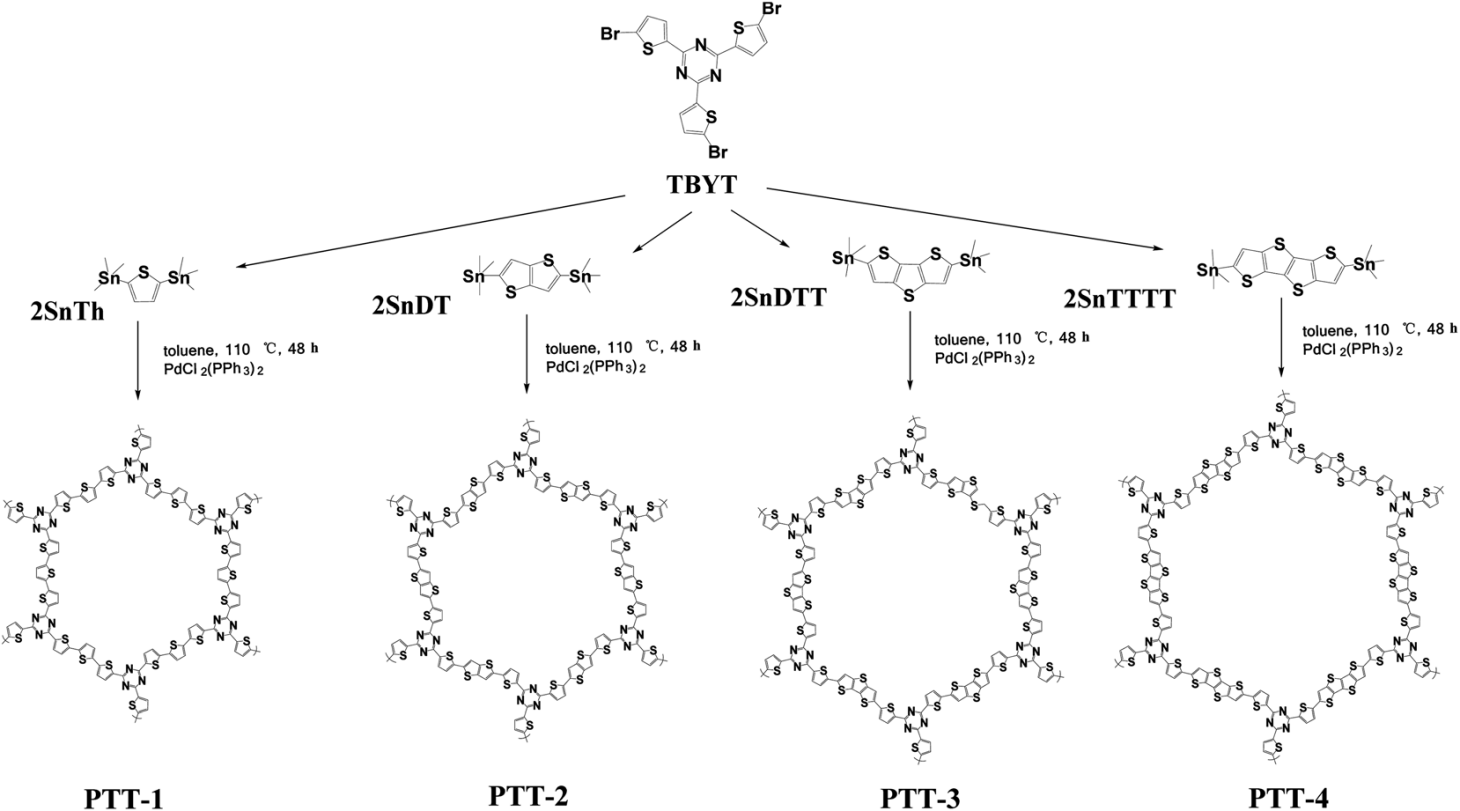
Schematic diagram of the synthesis of the polymers. (a–d) Synthesis of PTT-1, PTT-2, PTT-3, PTT-4 with the common monomer 2,4,6-tris(5-bromothiophen-2-yl)-1,3,5-triazine (TBYT) and another four different monomers including 2SnTh (PTT-1), 2SnTT (PTT-2), 2Sn (DTT) (PTT-3), 2SnTTTT (PTT-4), respectively.

In this work, we adopted a triazine-based molecule (2,4,6-tris(5-bromothiophen-2-yl)-1,3,5-triazine) as the acceptor unit (also as the building unit) and a series of different thiophene derivatives as the donor units for the construction of the three-dimensional D–A (donor–acceptor) type polymers by the Stille cross-coupling reaction, which are named PTT-1, PTT-2, PTT-3 and PTT-4, as shown in Fig. 1. The D–A design approach might lead to the formation of low band gap conjugated microporous polymers (CMPs) by the combination of lower-lying LUMO energy levels and higher-lying HOMO energy levels, which could benefit the polymers with stronger electron affinity and higher electron conductivity, and finally in turn enhance the electrochemical performance of the polymer as anode material. Then their composites with Vulcan XC-72 addictive carbon are prepared by the *in situ* polymerization method, which are used as the anode electrode materials in LIBs to test their electrochemical performances. It was also noticed that the linkage units of thiophene or fused cyclothiophene derivatives have a strong effect on the conjugation degree, the band gap and the morphologies of the as-prepared polymers. Accordingly, the optimized design of the molecules to obtain the low band gap, the cross-linked structures and higher electrochemical performances are then discussed in this work.

## 2. Experimental section

### 2.1. Materials

2,5-Bis(trimethylstannyl)thiophene (2SnTh); 2,5-bis(trimethylstannyl)thieno[3,2-*b*]thiophene (2SnTT); bisthieno[3,2-*b*:2′,3′-*d*]thiene-2,6-diylbis(trimethylstannane) (2SnDTT); 2,6-bis(trimethylstannyl)thieno[2′,3':4,5]thieno[3,2-*b*]thieno[2,3-*d*]thiophene (2SnTTTT) are purchased from SunaTech Inc. Bis(triphenylphosphine)palladium (PdCl_2_(PPh_3_)_2_), Vulcan XC-72 carbon, tetrabutylammonium hexafluorophosphate (TBAPF_6_, 98%), acetonitrile (ACN), 1-methyl-2-pyrrolidinone (NMP, 99.9%), ethanol, trifluoromethanesulfonic acid (TfOH), chloroform, methanol, acetone and toluene are bought from Aladdin Co., LTD. 5-Bromothiophene-2-carbonitrile is obtained from Alfachem (Zhengzhou, China). All reactants are of analytical grade and used without further purification.

### 2.2. Synthesis of TBYT

For the synthesis of TBYT, 3.6 g of TfOH was added to a solution of 5-bromothiophene-2-carbonitrile (4.6 g) in 300 mL of chloroform at 0 °C under electromagnetic stirring. Three hours later, the mixture are raised to room temperature, and then the mixture was stirred for another 45 hours. After that, the solvent was removed by rotary evaporation, and the residue was neutralized with 3 mL ammonium hydroxide (25% in water). The precipitate was obtained by filtration and be washed with excessive water, and then be dried in a vacuum oven overnight at 60 °C. The raw product was recrystallized in toluene, and the gray product was obtained by filtration and dried overnight in a vacuum at 60 °C. The ^1^H NMR and ^13^C NMR spectra of the TBYT were given in Fig. S1†.^20^

### 2.3. Synthesis PTT-4 and PTT-4@C

#### 2.3.1. Synthesis of PTT-4

Firstly, 300 mg of TBYT and 461.06 mg of 2BrTTTT were added into to a round-bottom flask, being added with 80 mL of toluene as the solvent and 60 mg of PdCl_2_(PPh_3_)_2_ as the catalyst. Then, the flask was kept in the oil-bath and the mixture was refluxed under 110 °C with magnetic stirring for 48 h under argon gas environment. After that, the solid product was obtained after filtration, and then was extracted with *n*-hexane, methanol, and acetone respectively, for 24 hours, to remove the impurities and catalysts. Finally, the samples were oven-dried at 80 °C for 4 h to get the final products.

#### 2.3.2. Synthesis of PTT-4@C

The composite material PTT-4@C was also obtained following the same procedures described above, with the additional addition of 872 mg of Vulcan XC-27 carbon powders as the support materials during the reaction process. After the reaction process, the raw products are subjected to the filtration, extraction and drying processes, and the final products are collected and used as the active materials in electrodes. It was calculated that the mass ratio of the polymer PTT-4 was 30% in the PTT-4@C composite. The synthesis of other polymers and composites as well as other experimental details were given in ESI.†

## 3. Result and discussion

### 3.1. Structural information

**Fig. 2:**
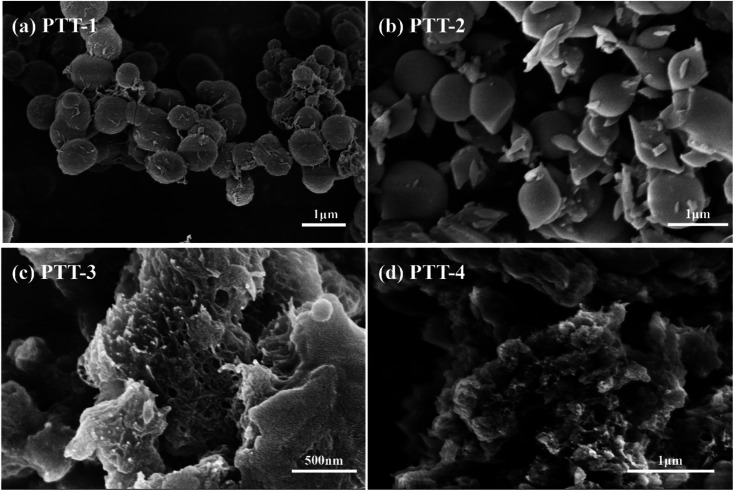
(a–d) SEM imagines of (a) PTT-1, (b) PTT-2, (c) PTT-3 and (d) PTT-4.

**Fig. 3:**
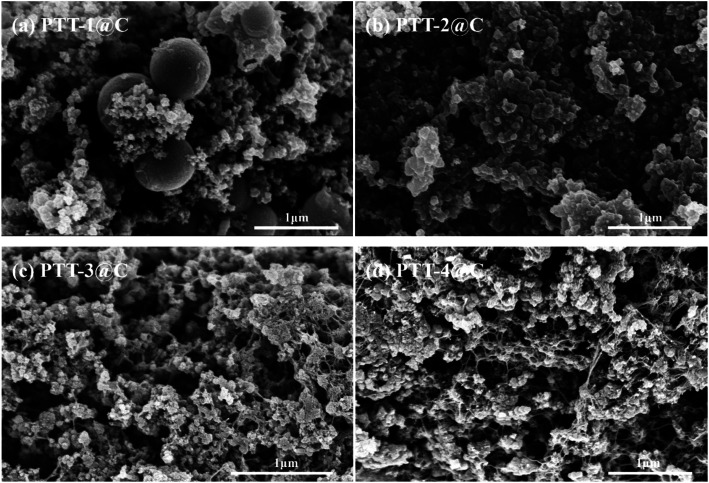
(a–d) SEM imagines of (a) PTT-1@C, (b) PTT-2@C, (c) PTT-3@C and (d) PTT-4@C.

Fig. 2 and 3 show the SEM images of the polymers and the composites respectively, the images of the pure Vulcan XC-72 carbon that used in the composites under small magnification are given in Fig. S2 and S3.† It can be seen from Fig. 2(a), the PTT-1 particles agglomerated together and formed regular sphere. After the in-suit polymerization with carbon materials, most of the PTT-1 particles retained their original sphere morphology and be embedded into the carbon matrix (Fig. 3(a)). Besides, the homogeneous element distribution in EDS elemental mapping analysis (Fig. S4†) also indicated that a portion of polymers also had been coated on the surface of the carbon particles. The PTT-2 particles became much more irregular than that of PTT-1, there are many irregular shaped particles in addition to a small number of spherical particles (Fig. 2(b)). It's hard to distinguish the polymer particles from the carbon particles in the image of the composite material PTT-2@C (Fig. 3(b)), but the uniform element distributions of S and N in elemental mapping indicated that the coating of the polymers on the surface of carbon particles also occurred. As for PTT-3 and PTT-4, a netlike and porous bulky structure could be seen in both Fig. 2(c) and (d), respectively. As these two polymers are formed *in situ* with the presence of the carbon powders, the small-sized carbon particles prevents the self agglomeration of the polymers, and instead, the polymers interlaced with the carbon particles together and formed a cross-linked netlike structure (Fig. 3(c) and (d)).

Different with the linkage unit Th or TT, the adoption of the longer linkage units like DTT and TTTT would be beneficial to reduce the steric hindrance effect within the conjugated microporous polymers (CMPs). Therefore, the polymers PTT-3 and PTT-4 were prevented from forming spherical or granular structures through self-aggregation, and as they composited with the carbon powders, a cross-linked netlike structure could be formed. The netlike structures of PTT-3@C and PTT-4@C make it easier for the penetrating of the electrolyte into the interior of the anode materials, therefore promote the transportation of ions and electrons and also provide more electro-active sites for the electrochemical reaction.

**Fig. 4:**
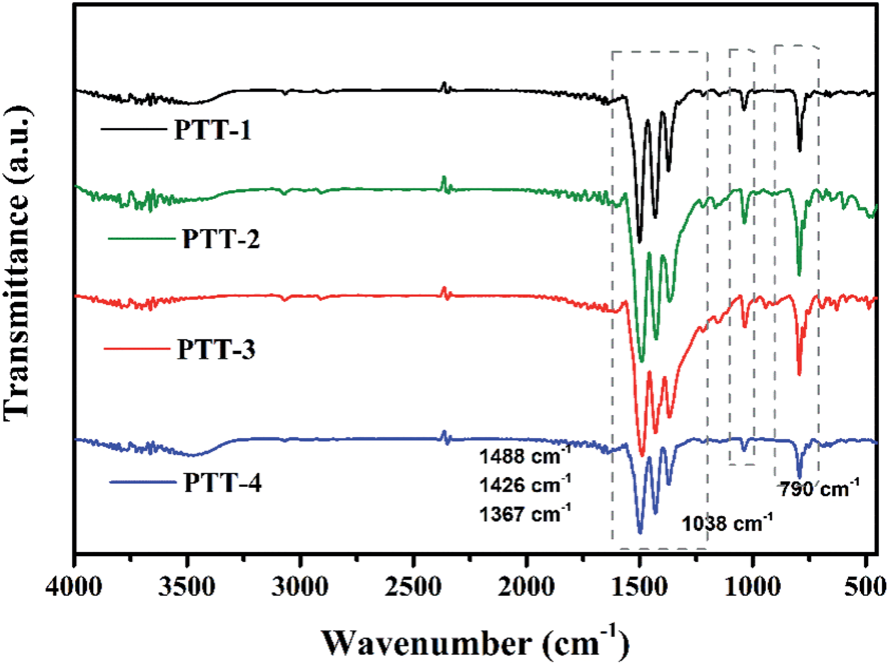
FT-IR spectrum of PTT-1, PTT-2, PTT-3 and PTT-4.

FT-IR was used to verify the structures of the polymers, and the results are shown in Fig. 4. Except the broad peak at around 3440 cm^−1^, which is caused by the O–H of trace water in the samples, the absorption peak at around 790 cm^−1^ can be assigned to the C_β_–H out-of-plane bending vibration of thiophene groups, while the peak at around 1038 cm^−1^ is the in-plane bending vibration of C_β_–H. Four characteristic peaks observed at around 1367–1633 cm^−1^ are attributed to the C=C, C=N skeletal vibration of triazine and thiophene groups, which is an important evidence of the existence of aromatic compound.^21^ And it can be noticed that with the increase of the conjugation length of the fused cyclothiophene derivatives, the peaks shift slightly to the low wave number gradually, which might due to the fact that the enhanced conjugated system would facilitate the uniform distribution of the electron cloud, therefore the bond strength as well as the vibration frequency of C=N and C=C reduced, thus causes the shift towards the low wave number direction.

**Fig. 5:**
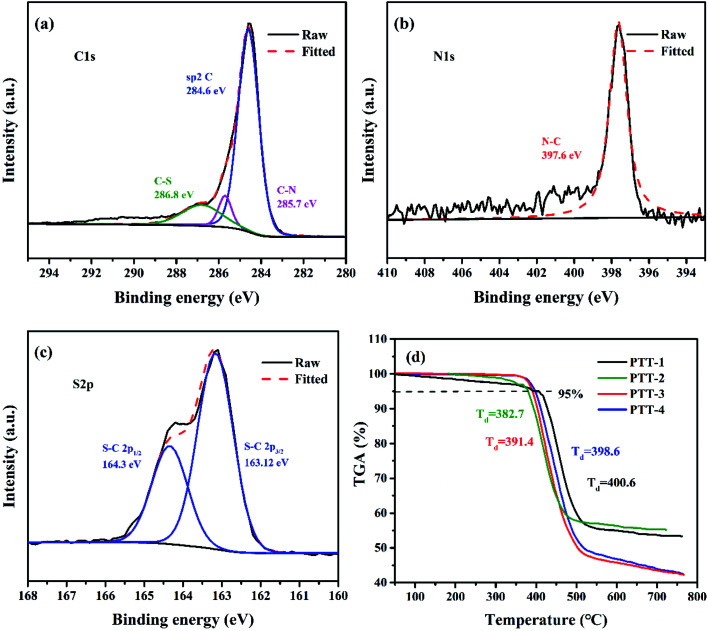
High-resolution XPS spectra of the composite PTT-4@C; (a) C 1s, (b) N 1s and (c) S 2p; (d) TGA curves of the pure polymers.

Fig. 5(a)–(c) shows the XPS high-resolution spectroscopy of C, N, S of the PTT-4@C composite, and the spectra for other composites are shown in Fig. S5–S7.† The separated peaks of C 1s at 284.6 eV, 285.7 eV and 286.8 eV belong to the sp^2^ C=C in the thiophene rings, the sp^2^ C–N in triazine groups and the sp^2^ C–S in thiophene rings respectively. The splitting peaks of S 2p including S 2p_1/2_, S 2p_3/2_ around 164 eV are the sp^2^ C–S in thiophene rings, and the N 1s peak at 397.8 eV is the pyridinic-N atom in triazine unit.^22^

Thermogravimetric analysis was taken to evaluate the heat resistance of the four pure polymers including PTT-1, PTT-2, PTT-3 and PTT-4(Fig. 5(d)). The initial decomposition temperature (*T*_d_) usually refers to the temperature at which the mass loss of the polymer reaches 5%. According to this, the *T*_d_ for PTT-1, PTT-2, PTT-3 and PTT-4 were 400.6 °C, 382.7 °C, 382.7 °C and 398.6 °C, respectively. All the polymers have high thermal stability above 380 °C, and the ultra-high thermal stability of the polymers can guarantee the safe-use of the anode materials in LIBs.

The pore structures of the composites were measured by nitrogen isotherm adsorption–desorption at 77.3 K. The curves for both the pure carbon powder and the composites are shown in Fig. S8.† Apparently, the profiles of the curves of the composites are similar to that of the pure Vulcan XC-72 carbon, which exhibit a combination profile with type IV isotherms and H3-type hysteresis loop. The appearance of the hysteresis loop under high relative pressure is due to the capillary condensation of the vapor happening in the slit pores in the composites, indicting formation of mesoporous pores due to the loose accumulation of polymer/carbon particles. The closure points of hysteresis loops of the four composites are 0.8, 0.8, 0.6 and 0.6 for PTT-1@C, PTT-2@C, PTT-3@C and PTT-4@C, respectively, with the latter two samples have lower closure points than that of the former two samples, suggesting the higher proportion of smaller pores (in mesoporous level) in PTT-3@C and PTT-4@C (Fig. S8(f)†), which may benefit from their netlike porous structure. The specific surface areas and the average pore sizes of the pure carbon and the four composites are given in Table 1. Apparently, the BET surface areas of all the composites are lower than that of the pure carbon material, which might due to that the formation of the composites results in the aggregation of particles and the reduction of the number of stacking pores (mainly macropores), and the surface coating process of the polymers on the carbon particles also covered a portion of original pores. In addition, the latter two composites PTT-3@C and PTT-4@C have higher specific surface areas than PTT-1@C and PTT-2@C, which may be attributed to their loose cross-linked structure.

**Table 1:** BET surface area and average pore size of the composites

	Pure carbon	PTT-1@C	PTT-2@C	PTT3@C	PTT-4@C
BET surface area (m^2^ g^−1^)	173.0722	79.2132	96.5028	114.4627	120.3353
Average pore size (nm)	18.3805	21.6674	18.4179	17.5994	19.3843

### 3.2. The energy levels of the donor–acceptor polymers

The band gaps (*E*_g_) of the four polymers were measured through UV-vis absorption spectroscopy. According to the Tauc plot method,^23,24^ the *E*_g_ of the polymers can be calculated by eqn (1).

**Fig. 6:**
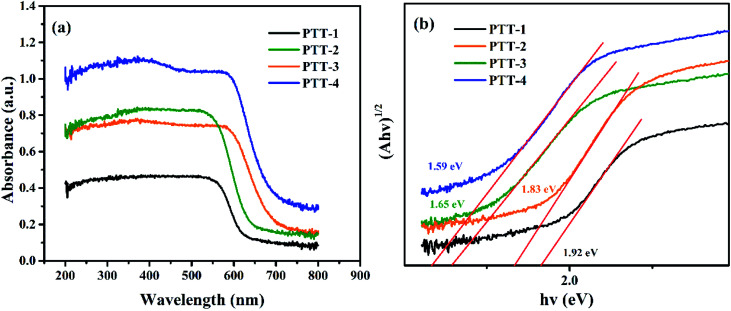
(a) The UV-vis spectra of the four polymers; (b) (*αhv*)^1/2^*vs. hv* curves of the four polymers.

1 (*αhv*)^1/2^ = *K*(*hv* − *E*_g_)

in which *α* is the absorption coefficient, *h* is the Planck constant, *v* is the vibration frequency and *K* is a constant. The plot of (*αhv*)^1/2^ against *hv* was made and the linear part of the plot is extrapolated to the *X*-axis, and the intercept was the *E*_g_ values of the polymers. Fig. 6 shows the UV-vis absorption spectra and the Tauc plot of the polymers. The *E*_g_ of the polymers are measured to be 1.92 eV, 1.83 eV, 1.65 eV and 1.59 eV, for PTT-1, PTT-2, PTT-3 and PTT-4 respectively, a continuous decrease in *E*_g_ values is observed as the enlargement of the molecular structure of the fused thiophene derivatives. This is because the planarity of the thiophene derivatives is increased with the increase number of fused thiophene rings, and this can bring the molecule a higher conjugation degree as well as a stronger electron donating ability. For the triazine unit has an electron deficient property as combined within an organic framework, the stronger interaction between the triazine units and fused thiophene rings with strong electron donating abilities could further decrease the *E*_g_ of the polymers. The low band gap is conducive to the delocalization of the π electrons along the organic frameworks, which improves the electron conductivity and stabilizes the intercalated lithium ions during the discharge process.^26,27^ Then the HOMO/LUMO energy levels of the polymers are also measured and calculated by the CV method, the details were given in Fig. S9.† The LUMO (and HOMO) energy levels are −3.56 eV (−5.48 eV), −3.55 eV (−5.38 eV), −3.63 eV (−5.31 eV), −3.59 eV (−5.18 eV), respectively, for PTT-1, PTT-2, PTT-3 and PTT-4. The HOMO energy levels are from PTT-1 to PTT-4, the HOMO energy levels of the polymers are elevated by introducing the more enlarged fused thiophene derivatives, which means the gradually enhanced reduction ability of the polymers for the p-type doping during the charging process, and therefore could accommodate more electrolyte ions within the polymers to provide higher specific capacity during the charge–discharge process.^28^ The polymers reported in this study have slightly higher *E*_g_ values to that of the state of the art polymer (AzoBT) newly reported by Jiang *et al.*, but they have lower LUMO energy levels that of AzoBT, indicating that the polymers in this study have stronger abilities for electron affinity in the discharge process.^16,25^

### 3.3 Electrochemical properties

**Fig. 7:**
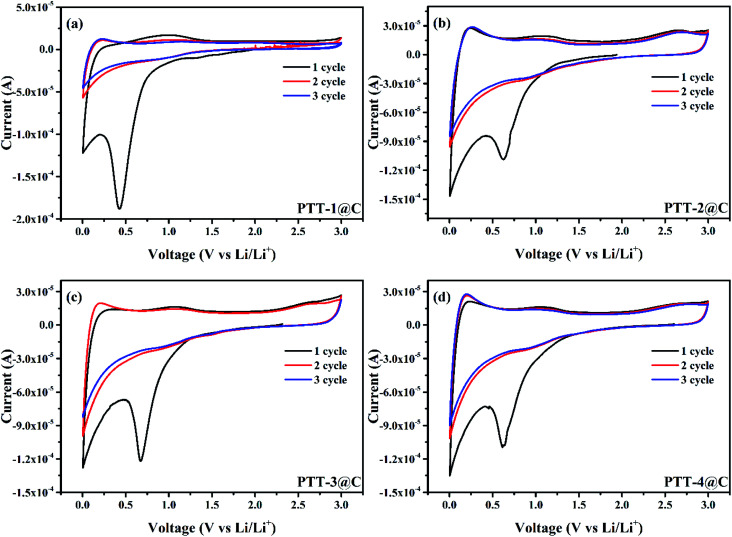
CV cures of (a) PTT-1@C, (b) PTT-2@C, (c) PTT-3@C, (d) PTT-4@C with scan rate of 1 mV s^−1^.

Fig. 7 shows the cyclic voltammetry (CV) curves of the composites in a coin-type battery with the scan rate of 0.1 mV s^−1^, and the scan voltage difference was from 0.005 V to 3 V (*vs.* Li/Li^+^). Two obvious reduction peaks at around 0.5 V and 0 V can be observed during the first cathodic scan for all the materials, corresponding to the formation process of the SEI (solid electrolyte interphase) film on the surface of the electrode, which is caused by the decomposition of the electrolyte that can be seen in most of the anode materials.^29^ And these two peaks disappeared in the next several cycles, indicting the SEI film was completely formed in the first discharge process and didn't engage in the later reactions. Changing to the anodic scan, the n-type undoping process would occur, in which the Li^+^ ion left the composites and migrated into the electrolyte, and an oxidation peak was observed at around 1 V (the first scan), corresponding to the re-oxidation (n-type undoping) of the polymers (For all polymers). The intercalation/deintercalation potentials of Li^+^ ions were found at around 0 V and 1 V, respectively, for the carbon matrix and the polymers. From the CV curves of PTT-2@C, PTT-3@C and PTT-4@C, there all have a small oxidation peak at around 2.5 V, which refers to the p-type doping process after all the Li^+^ ions had been deintercalated from the composites.^30^ In this process, the PF_6_^−^ in the electrolyte migrated into the polymer's matrix and form the p-type doping state [*A*^+^*x*PF_6_^−^], where *A* refers to the polymer's matrix, and *x* refers to the doping levels of the polymers.^28^ The relatively low n-type doping redox potentials *vs.* Li/Li^+^ here of the four composites (around 1 V) suggest that the prepared composites in this work can be used as the anode materials in LIBs.

### 3.4. The capacitance of the composites

**Fig. 8:**
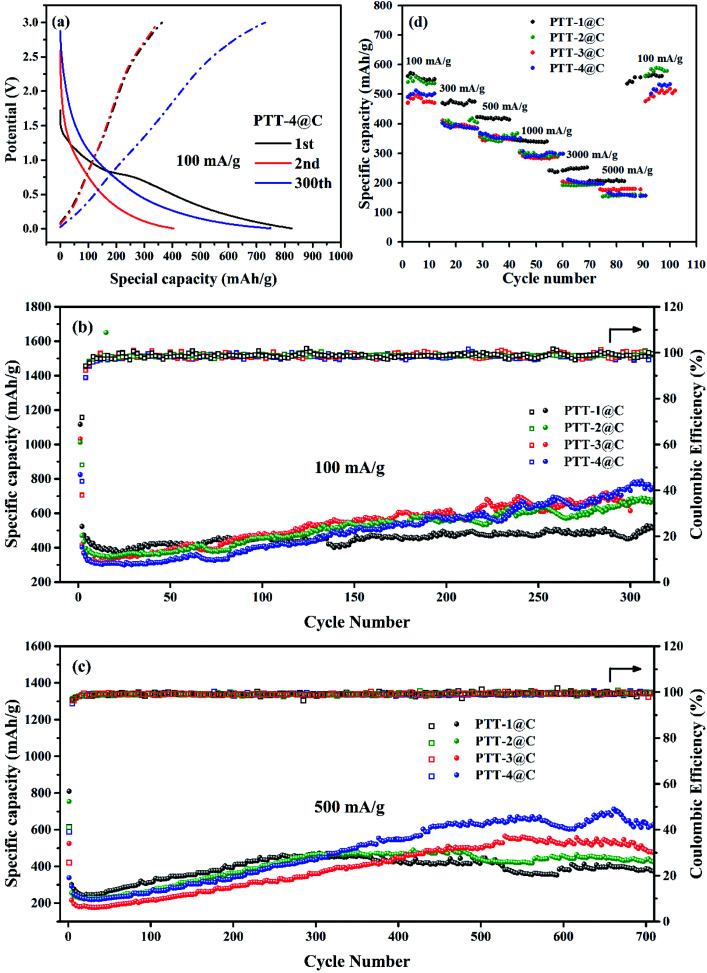
(a) GDC curve of PTT-4@C at different cycles, 100 mA g^−1^. (b and c) Constant current discharge curve of PTT-1@C, PTT-2@C, PTT-3@C and PTT-4C at 100 mA g^−1^ and 500 mA g^−1^. (d) Rate capacity properties of PTT-1@C, PTT-2@C, PTT-3@C and PTT-4C at different current density.

The charge and discharge test of the prepared electrodes were carried out with the potential range from 0.05–3.0 V *vs.* Li^+^/Li. Fig. 8(a) shows the galvanostatic charge–discharge (GCD) curve of PTT-4@C at 100 mA g^−1^ at different cycles. The data for other materials are shown in Fig. S10.† As shown in Fig. 8(a), a considerable decrease in capacity that caused by the formation of SEI film between the first and the second discharge can be noticed, and the two discharge plateau on the first discharge curve right accorded with the two cathodic peaks on the CV curves. Another plateau on the charge curves at around 2.5 V refers to the p-type doping process on the electrode, which can also be seen from the CV curves. The absence of the discharge plateau in the later cycles indicates that there is no obvious phase transition in the discharge process, the lithium intercalation process is a step-by-step reaction process and the redox potential of lithium intercalated polymer changes with the number of lithium ions introduced.^31,32^ For all the composites, the discharge process proceeded steadily to the discharge potential approached 0.05 V, which illustrated the good stability of the materials.

The specific capacities as well as the cyclic stability of the materials were examined at different current densities. Fig. 8(b) shows the cycling performances of the four materials at a current density of 100 mA g^−1^. The capacity fading after the first cycle brought by the formation of SEI film could be seen clearly for all the materials, which also led to the low coulombic efficiency of the first cycle. The initial decrease in capacity was terminated after about twenty-five cycles, and then the capacities of all the materials experienced a process of rising gradually, which could be found in many polymer electrodes, for some active sites are covered inside the polymers, and with the insertion and desertion of lithium ions, the active sites are then exposed and released gradually.^33^ The capacity increase might also be due to a structural relaxation process in the anode materials, during which with the increased penetration of electrolyte more active sites become accessible to the redox processes. Except for the initial several cycles, the coulombic efficiencies of the four composites were nearly 100%, proving the good cycling stability of the materials. It was noted that during the first 50 cycles, the order of the capacity is PTT-1@C > PTT-2@C > PTT-3@C > PTT-4, and in the next cycles, the above order reversed with the increase of the cycling number, and at the 300^th^ cycle, PTT-4@C had the highest capacity (772 mA h g^−1^), followed successively by PTT-3@C (707 mA h g^−1^) and PTT-2@C (671 mA h g^−1^), with PTT-1@C had the lowest capacity (495 mA h g^−1^). For the polymer-based electrodes, considering that some of the active sites within the materials are exposed gradually, this reversal can be explained reasonably by the differences among their structures.^34,35^ According to the data from SEM analysis, the composites of PTT-3@C and PTT-4@C had a porous cross-linked structure, and as a resultant these two composites had higher specific surface areas than the other two composites. The above structural characteristics enabled PTT-3@C and PTT-4@C to have more potential active sites to accommodate Li^+^ than the former two composites, and the structures of the polymers were relaxed with the embedded active sites were thereto released as the charge and discharge processes.

The cyclic performances of the composite materials were also evaluated at a higher current density 500 mA g^−1^ (Fig. 8(c)). After a rapid decline and a slow rise processes, the specific capacities of PTT-1@C and PTT-2@C began to decline slowly after about 470 cycles, whereas the composites PTT-3@C and PTT-4@C had a longer period of capacity increasing stage up to 550 cycle, and then entered into a stable period until 700 cycle or even more. At around 680^th^ cycle, the composite materials could retain the capacities of 365 mA h g^−1^, 424 mA h g^−1^, 561 mA h g^−1^ and 660 mA h g^−1^ at the 500 mA g^−1^, for PTT-1@C, PTT-2@C, PTT-3@C and PTT-4@C, respectively. Apparently, the latter two composites showed higher capacities than the former two, which was in accordance with the data under 100 mA g^−1^. In this case, the porous cross-linked structure is beneficial to increase the specific capacity and the stability of the polymer-based electrode materials.^36^Fig. 8(d) showed the rate capacities of the composite materials with the current densities changed in turn, including 100 mA g^−1^, 300 mA g^−1^, 500 mA g^−1^, 1000 mA g^−1^, 3000 mA g^−1^ and 5000 mA g^−1^. It could be noticed that all the composites had the ability to recover from the decreased capacity caused by the immediate increased current.

### 3.5. The EIS measurement

**Fig. 9:**
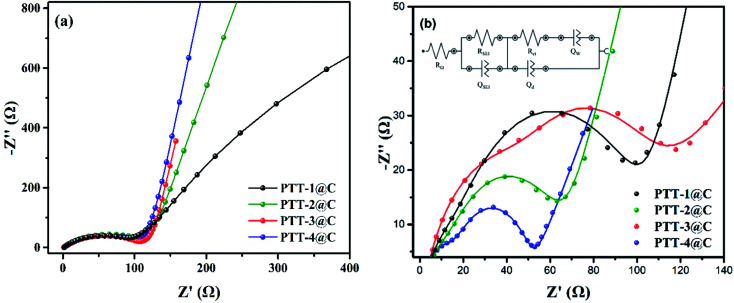
EIS spectra of the composites at (a) open-circuit voltage, (b) polarization potential.

The EIS was measured from 100 000 Hz to 0.01 Hz for the polymer-based electrodes to examine the electrical behavior of the composites. The Nyquist plots of the composite electrodes are shown in Fig. 9, where a typical impedance spectrum of the insertion-host type electrode materials can be seen. Fig. 9(a) is the EIS spectrum under the open-circuit voltage. The arc in high frequency region corresponds to the diffusion and migration process of Li^+^ on the surface of the electrode materials. The arc in the low and middle frequency corresponds to the charging and discharging processes of the double-layer capacitance. And because the insertion/desertion processes of Li^+^ in electrodes hadn't occurred at the open-circuit voltage, there was a big capacitive arc (represented the high charge transfer resistance) in low and middle frequency. When the test voltage was set at the polarization potential, as shown in Fig. 9(b), the Li^+^ would begin to transfer throughout the electrodes, and under this condition the spectrum is constituted by two arcs and a straight line of which the slope is close to 1. The first arc in high frequency refers to the transportation of Li^+^ in SEI film, the other arc is the transportation process occurring on the interface between SEI film and electrode. The line refers to the diffusion-controlled process, which is caused by the so-called Warburg impedance. In addition, the Nyquist plots were then fitted to the equivalent circuit model in order to analyze the properties of the four polymer-based electrodes qualitatively (inset in Fig. 8(b)). The *R*_Ω_ refers to the ohmic resistance of the cell, including the resistance of the electrolyte and the electrode itself, *Q*_SEI_ and *R*_SEI_ are the capacitance and resistance of the SEI film, respectively. *Q*_d_ is the double-layer capacitance, *R*_ct_ is the charge transfer resistance and *Q*_w_ is the diffusion impedance.^37,38^ Considering most of the parameters in the circuit are highly related to the different type of electrolytes and the separators, *R*_ct_ is always used to evaluate the electrical conductivity of the electrode itself. According to the simulated circuit, the *R*_ct_ of PTT-1@C, PTT-2@C, PTT-3@C and PTT-4@C are 91 Ω, 63 Ω, 56 Ω and 36 Ω respectively. Since *R*_ct_ is used to measure the charge transfer ability in the electric double layer between the surface of the electrode and the SEI film, it is reasonable to deduce from the result that the materials with net-like frameworks (PTT-3@C and PTT-4@C) could promote the transportation of electrons and ions, which revealed the better electrochemical performances of the latter two materials.

### 3.6. The capacities of polymers and their structure–activity relationship

Since the weak conductivity of the polymers made it inappropriate to be used as the electrode material directly, thus the composite materials consisting of the pure polymers and the Vulcan X-72 carbon were synthesized in this work. For comparison purpose, a blank control using only carbon powder as the electrode was also tested under the same condition, and the specific capacity the electrode was found at around 154 mA h g^−1^ under 100 mA g^−1^ (Fig. S11†). Subsequently, the performances of the pure polymers can be evaluated by horizontal comparison. The specific capacities of PTT-1@C, PTT-2@C, PTT-3@C and PTT-4@C under 100 mA g^−1^ were 495 mA h g^−1^, 671 mA h g^−1^, 707 mA h g^−1^ and 772 mA h g^−1^, respectively. From PTT-1 to PTT-4, the band gaps of the organic frameworks decreased with the increased sizes of the fused thiophene rings in the polymers. The narrowed band gaps as well as the leveled up HOMO energy levels can improve the electronic conductivity of the polymers and facilitate the smooth migration of the π-electrons along the polymer's backbone and stabilize the intercalated lithium ions more effectively, which is conducive to the introduction of the subsequent lithium ions to the polymer matrix. Besides, with introduction of the fused longer thiophene derivatives in the polymers, the composites are tend to form cross-linked structures with higher specific surface areas, which is attributed to the decreased steric hindrance effect. And the cross linked network structure found in PTT-3@C and PTT-4@C is superior to the simple accumulation morphology for PTT-1@C and PTT-2@C. To further verify our conclusions, two other polymers and their composites with Vulcan XC-72 carbon, which were named as PTT-5, PTT-6, PTT-5@ and PTT-6@C were also synthesized and tested, as shown in Fig. S12.† Compared with the fused thiophene derivatives, the bithiophene and terthiophene groups in PTT-5 and PTT-6 have longer molecular chains and smaller steric hindrance effect, since the two adjacent thiophene rings could rotate freely and thus avoids the torsional strain. As a result, a clearly porous cross-linked structure can be seen in the SEM imagines of PTT-5@C and PTT-6@C (Fig. S13†). However, the rotation of the thiophene rings destroys the planarity of the molecule, for which decreases the conjugation degree of the thiophene derivatives, and makes the *E*_g_ a bit higher than the fused rings, and the values are 1.81 and 1.69 eV (Fig. S14†). The charge–discharge test result is given in Fig. S15,† and the specific capacity of PTT-5@C and PTT-6@C are 652 mA h g^−1^ and 725 mA h g^−1^, further illustrates that the specific capacity increases with the increased thiophene contents in the molecule. From the experimental results we could draw the conclusion that the synergistic effect of lowered band gap, the cross-linked structures as well as the higher thiophene contents of the materials could improve the specific capacity of the electrode materials. Among them, the cross-linked structures of the polymers could be constructed by using the monomers with long molecular chains to decrease the steric hindrance effect along the conjugated microporous polymers.

## 4 Conclusion

In summary, we synthesized four CMPs based on thiophene armed triazine unit and different thiophene derivatives, and also prepared the composites between the CMPs and addictive carbon by the *in situ* polymerization method. With the increase of the molecular size of the linkage group, it is easier for the composites to form a cross-linked structure. In this case, the two latter composites PTT-3@C and PTT-4@C have more homogeneous morphologies than PTT-1@C and PTT-2@C, which might be attributable to the relaxed intramolecular strain derived from the introduction of the longer linkage units. At a resultant, the latter two composites have higher specific areas than that of the former two composites as a resultant of the formation of the crosslinked porous structure. Due to the higher planarity and conjugation length, PTT-3 and PTT-4 have lower band gaps than that of the former two CMPs. With the synergy of these factors, PTT-3@C and PTT-4@C possessed superior performances to that of the former two composites, such as higher capacity and higher stability. In all, the successful construction of CMPs based on thiophene-armed triazine proved their effectiveness in adjusting the band gaps and morphologies of the polymers by changing the molecular sizes of the linkage units, and the strategy was also efficient in tuning the structures of the composites formed between the CMPs and carbon material. The CMPs showed good performances as the anode materials in LIBs and provided useful information for the molecular design of the polymer based anode materials as well as for the construction of composites between CMPs and carbon.

## Author contributions

Writing-original draft preparation, X. Xue; software and data process, J. M. Luo; polymer synthesis, L. Q. Kong; writing-review and editing, J. S. Zhao and Y. Xie; methodology, Y. Zhang; the electrochemical performance of the batteries, H. M. Du; supervision, S. Chen. All authors have read and agreed to the published version.

## Data availability statement

Data availability upon request.

## Conflicts of interest

No any competing interest involved.

## Supplementary Material

RA-011-D0RA10862F-s001
