# Supplementary material for: The synthesis of triazine–thiophene–thiophene conjugated porous polymers and their composites with carbon as anode materials in lithium-ion batteries
Source: RSC Adv. 2021 Mar 11;11(18):10688–98. doi: 10.1039/d0ra10862f (PMC8695664; doi:10.1039/d0ra10862f)
Supplement: RA-011-D0RA10862F-s001 [file RA-011-D0RA10862F-s001.pdf]

Electronic Supplementary Material (ESI) for RSC Advances.
This journal is © The Royal Society of Chemistry 2021

Supporting Information

The synthesis of triazine-thiophene-thiophenes conjugated porous polymers and their composites with carbon as anode materials in lithium-ion batteries

Xin Xue[a,b], Junming Luo[c], Lingqian Kong[d], Jinsheng Zhao[b,*], Yan Zhang[b], Hongmei Du[b], Shuang Chen[a,*], Yu Xie[c,*]

[a] *State Key Laboratory of Heavy Oil Processing, College of Chemical Engineering, China University of Petroleum (East China), QingDao, 266580, P.R. China*
[b] *College of Chemistry and Chemical Engineering, Liaocheng University, Liaocheng, 252059, P.R. China*
[c] *School of Environmental and Chemical Engineering, Nanchang Hangkong University, Nanchang 330063, China*
[d] *Dongchang Colledge, Liaocheng University, Liaocheng, 252059, P.R. China*

*Corresponding author.
E-mail address: j.s.zhao@163.com (j.s.zhao); chsh1030@163.com (S. Chen); xieyu_121@163.com (Y. Xie)

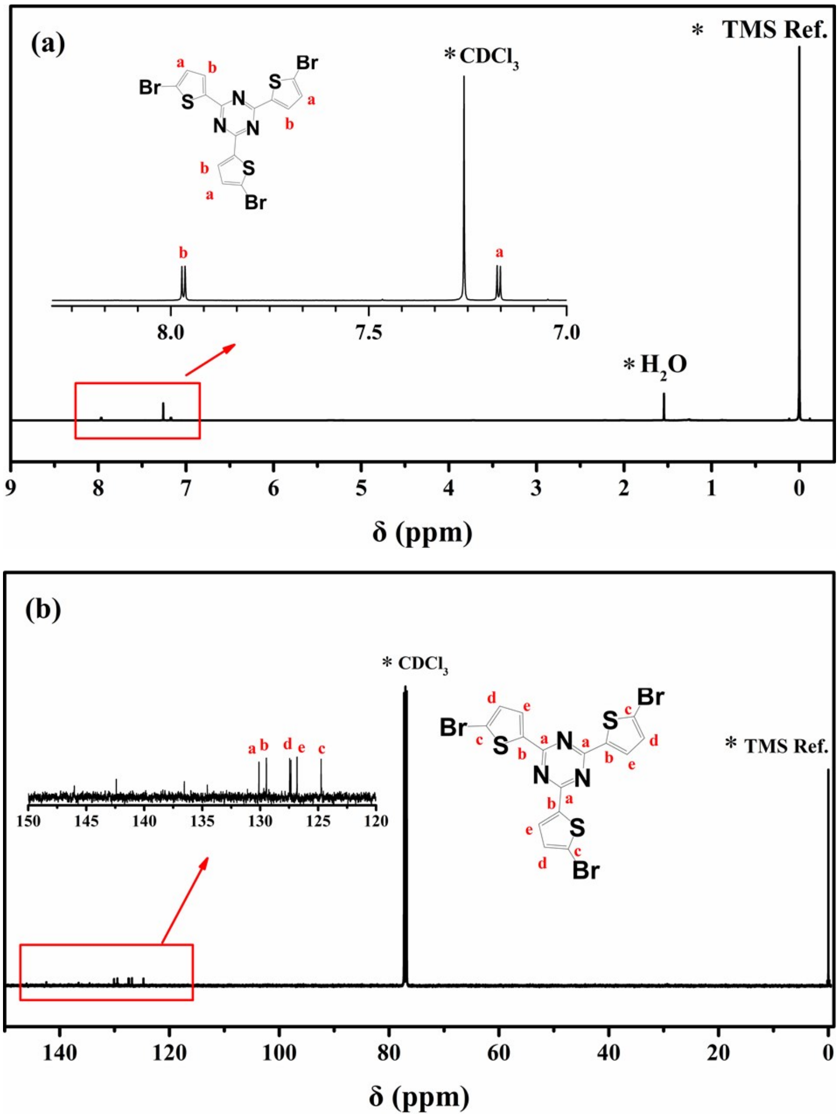

^{1}H NMR (500 MHz, Chloroform-*d*) δ 7.97 (d, *J* = 4.0 Hz, 3H), 7.17 (d, *J* = 4.0 Hz, 3H).

^{13}C NMR (126 MHz, CDCl3) δ 129.47, 127.46, 127.35, 126.82, 124.74.

Fig. S1. (a) ^{1}H NMR spectrum of TBYT, (b) ^{13}C NMR spectrum of TBYT.

Experiment:

Synthesis of PTT-1 and PTT-1@C:

300 mg 2,4,6-tris (5-bromothiophen-2-yl)-1,3,5-triazine(TBYT), 326.85 mg 2,5-bis(trimethylstannyl)thiophene (2SnTh) and 60 mg Bis(triphenylphosphine)palladium ($PdCl_2(PPh_3)_2$) catalyst were add to a round-bottom flask, with 80 ml methylbenzene as the solvent. Then, the mixtures were put into the oil-bath heating devices and refluxed under 110 ℃ with stirring for 48 h in Ar gas environment. After that, the solid products were obtained after filtration, and then were extracted with n-hexane, methanol, and acetone for 24 hours respectively to remove the impurities and catalysts. Finally, the samples were oven-dried at 80 ℃ for 4 h to get the final products.

The composite material PTT-1@C was also obtained from the same procedures described above, with additional 558.8 mg VulcanXC-27 carbon powders adding into the mixtures during the reaction process. It was calculated that the mass ratio of the polymer PTT-1 was 30% in the PTT-1@C composite.

Synthesis of PTT-2 and PTT-2@C:

300 mg 2,4,6-tris (5-bromothiophen-2-yl)-1,3,5-triazine(TBYT), 465.84 mg 2,5-bis(trimethylstannyl)thieno[3,2-b]thiophene (2SnTT) and 60 mg Bis(triphenylphosphine)palladium ($PdCl_2(PPh_3)_2$) catalyst were added to the round-bottom flask, the following produces are the same as the synthesis of PTT-1. For the synthesis of PTT-2@C composites, the mass of the Vulcan X-72 carbon was adjusted to 663 mg, and the mass ratio of the polymer in the composite was 30%.

Synthesis of PTT-3 and PTT-3@C:

300 mg 2,4,6-tris (5-bromothiophen-2-yl)-1,3,5-triazine(TBYT), 416.32 mg Bisthieno[3,2-

b:2',3'-d]thiene-2,6-diylbis(trimethylstannane) (2SnDTT) and 60 mg Bis(triphenylphosphine)palladium ($PdCl_2(PPh_3)_2$) were used during the synthesis process, others procedures are the same as PTT-1. For the synthesis of PTT-3@C, the mass of the Vulcan X-72 carbon was 767.6 mg, and the mass ratio of the polymer in the composite was 30%.

Structural characterizations:

The Fourier Transform Infrared spectroscopy were recorded on a Nicolet Avatar 360 FT-IR spectrometer with KBr pellets. The UV-Vis absorption spectroscopy were tested via Shimadzu UV-2550 spectrophotometer. The morphologies of the samples were observed through Hitachi Su-70 scanning electron microscopy (SEM, Hitachi Inc., Tokyo, Japan). The transmission electron microscopy (TEM) was examined to investigate the structural characterization using JEM-2100. The specific surface areas and porosity properties was examined by Nitrogen isotherm adsorption-desorption at 77.3 K using ASAP 2460-3 (Micromeritics) volumetric adsorption analyzer. X-ray photoelectron spectroscopy (XPS) was conducted with ESCALAB 250Xi spectrometer. X-ray diffraction (XRD) was carried out with the 2θ range from 5 to 800 using Kigaka D/max 2500 X-ray advance diffractometer with a Cu-Ka radiation, and a step scan mode was adopted with a scanning step of 0.02. The thermogravimetric analysis of the samples were conducted on a Netzsch STA449C TG/DSC thermal analyzer under nitrogen atmosphere between 20 °C and 800 °C.

Electrochemical measurements:

The electrochemical performances of the anode composites were tested with CR2032-type coin cells. A mixture is obtained by mixing the active material, acetylene black and polyvinylidene fluoride (PVDF) at a mass ratio of 6: 2.5: 1.5. The right amount of NMP was added to the mixture,

be grinded thoroughly to form a homogeneous slurry, and the slurry was then coated on copper foils. The coated copper foil was dried at 60 °C for 24 h, and then be cut into slices as the working electrode (anode for LIBs). The slices are further dried in vacuum drier at 120 °C for 8 hours, the constant weight of a slice was about 9 mg with the diameter of 12 nm. Then the as-prepared electrode was paired with Li foil as the counter electrode and the half battery was assembled in an argon-filled glove box. The electrolyte used was the solution of 1 M $LiPF_6$ dissolved in ethylene carbonate (EC) and dimethyl carbonate (DMC) (1:1 v/v) mixture. Galvanostatic charge-discharge experiments and rate capability were carried out on a land battery testing system (Land CT2001A, Wuhan, China) at room temperature. The same configured cells were also subject to cyclic voltammetry (CV) and electrochemical impedance spectroscopy (EIS) measurements on a potentiostat (PGSTAT 128N, Metrohm).

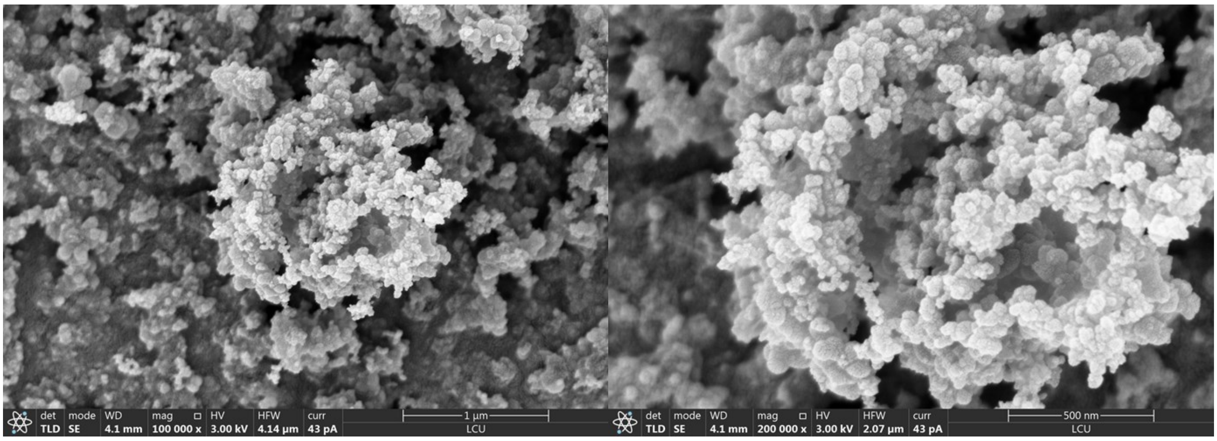

Fig. S2. SEM image of Vulcan XC carbon.

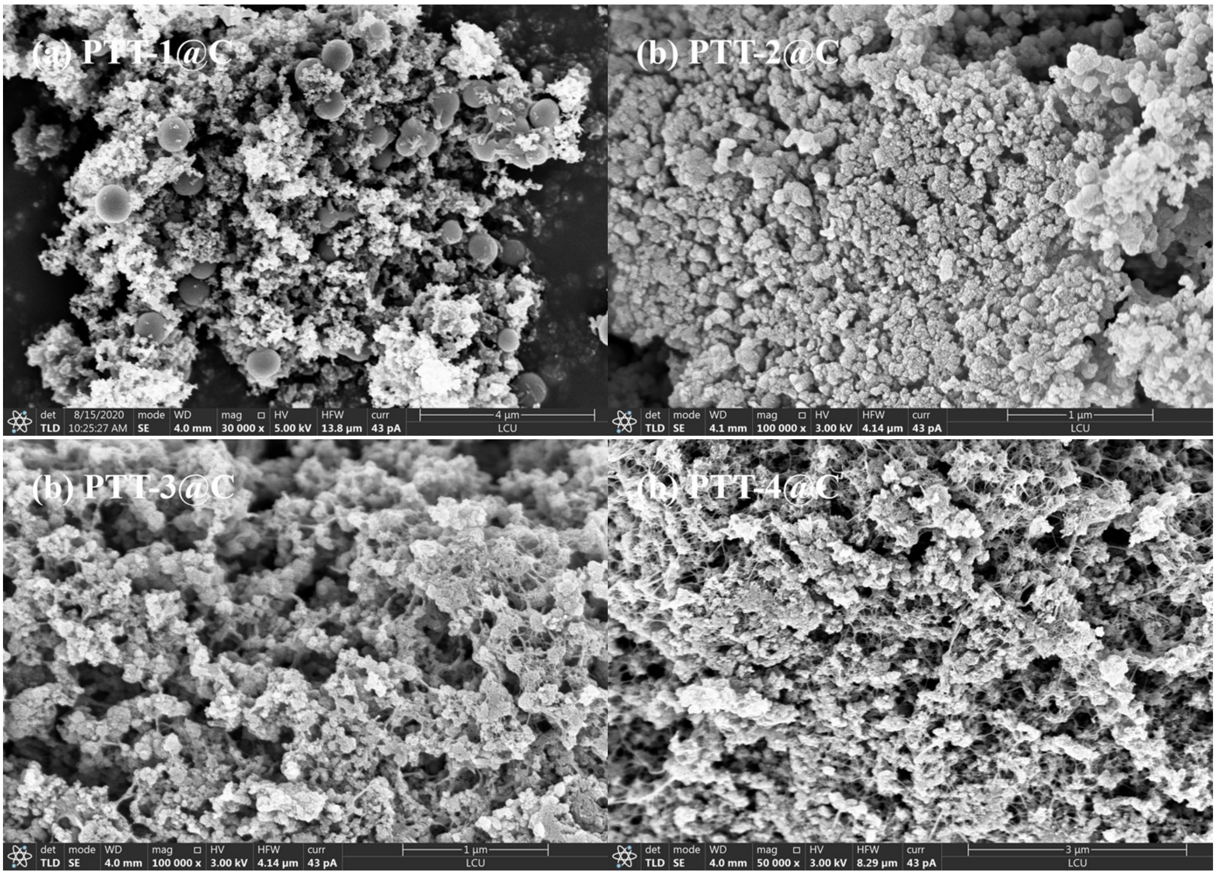

Fig. S3. The SEM imagines of PTT-1@C, PTT-2@C, PTT-3@C and PTT-4@C with small magnification.

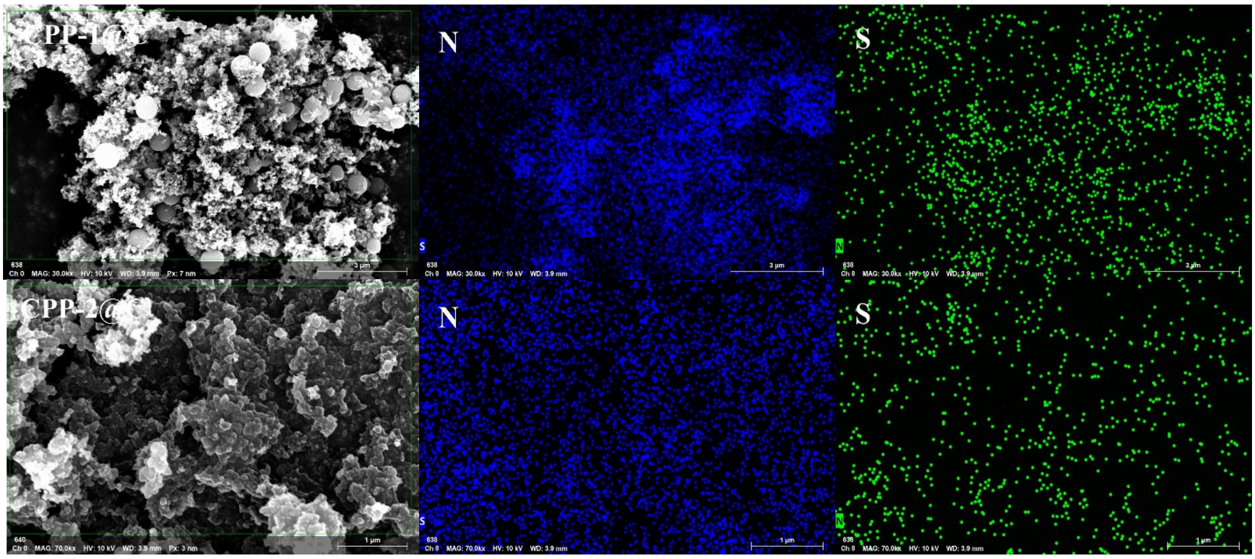

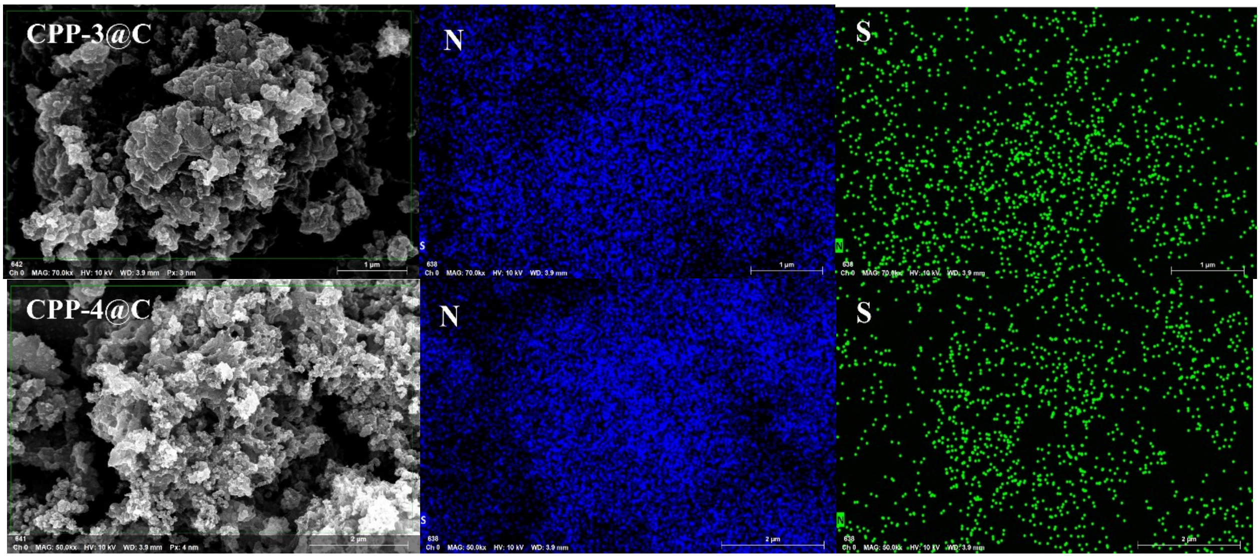

Fig. S4. Element mapping images for PTT-1@C, PTT-2@C, PTT-3@C and PTT-4@C from top to the bottom.

XPS analysis for PTT-1@C, PTT-2@C and PTT-3@C

PTT-1@C

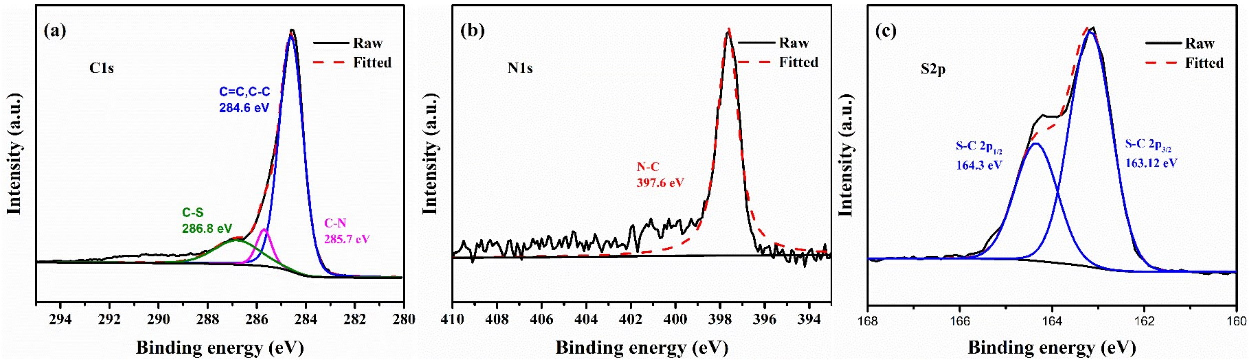

Fig. S5. High-resolution XPS spectra of the composite PTT-1@C; (a) C 1s, (b) N 1s, (c) S 2p.

PTT-2@C

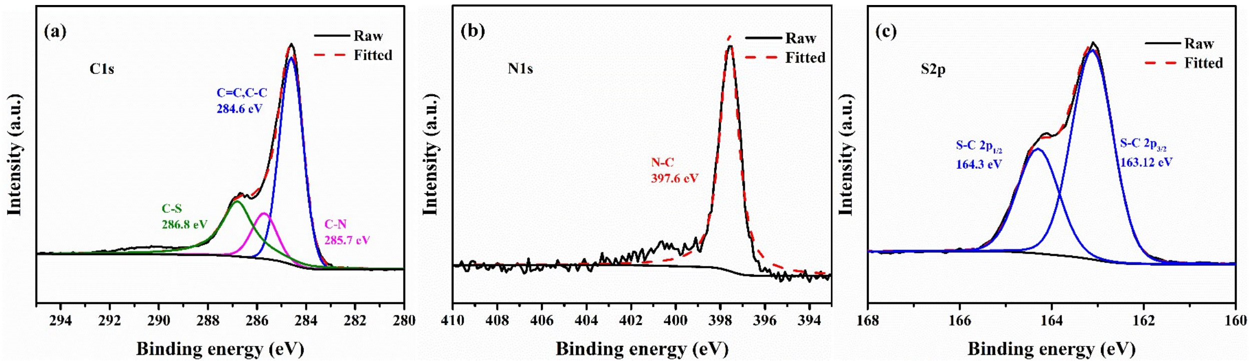

Fig. S6. High-resolution XPS spectra of the composite PTT-2@C; (a) C 1s, (b) N 1s, (c) S 2p.

PTT-3@C

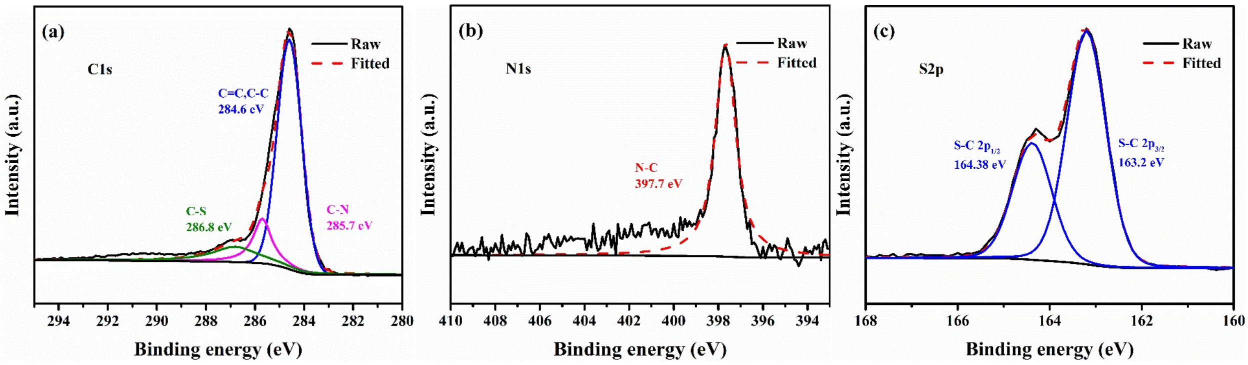

Fig. S7. High-resolution XPS spectra of the composite PTT-3@C; (a) C 1s, (b) N 1s, (c) S 2p;

Nitrogen adsorption-desorption isotherms of the composites

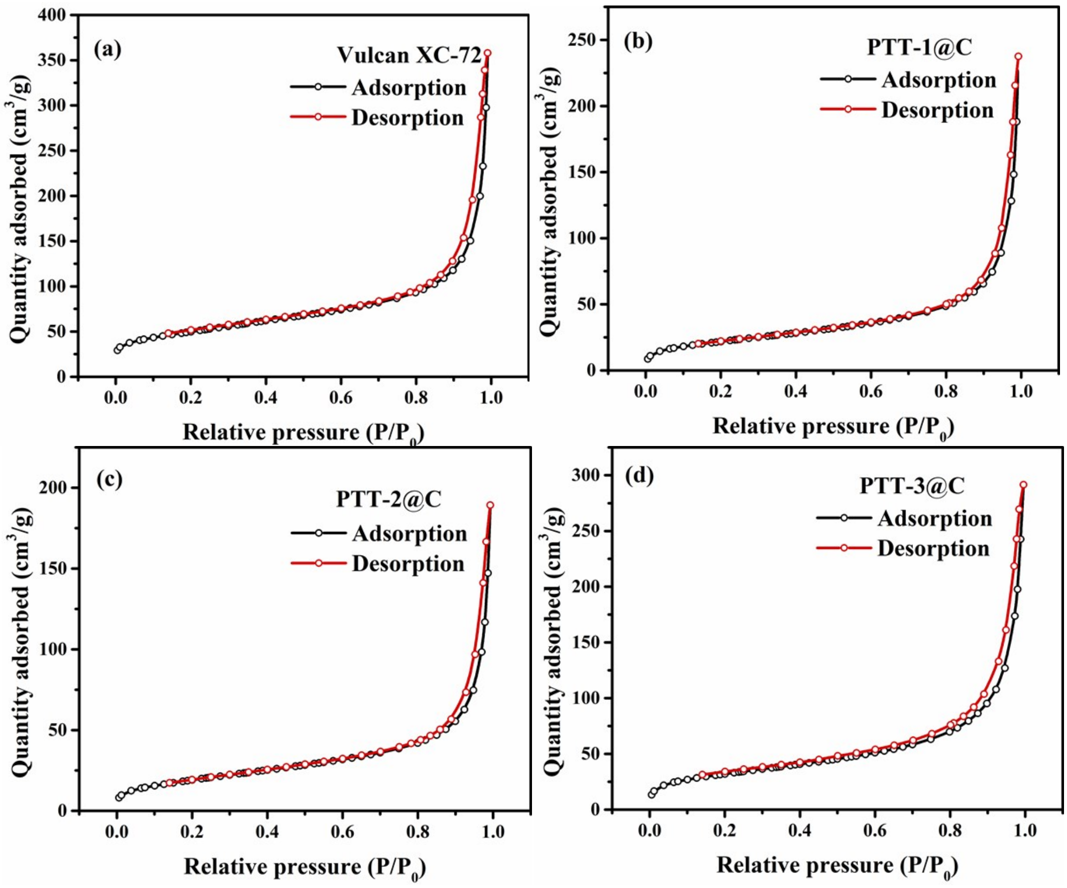

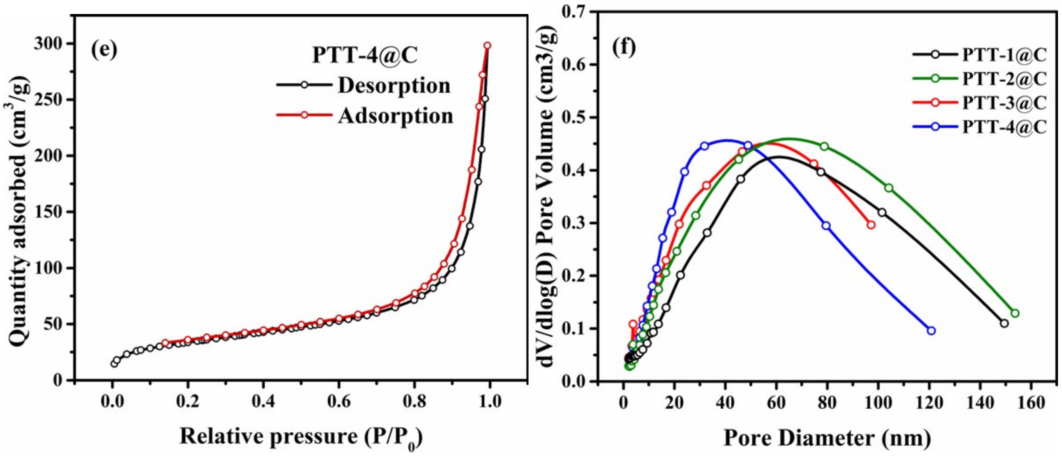

Fig. S8. Nitrogen adsorption-desorption isotherms of (a) Vulcan XC-72 carbon, (b) PTT-1@C, (c) PTT-2@C, (d) PTT-3@C and (e) PTT-4@C; (f) pore size distribution of the composites.

Cyclic voltammogram of the polymers

Cyclic voltammetry (CV) measurements of the polymers were carried out on a CHI660E (Chenhua, Shanghai) electrochemical workstation in a three-electrode-cell system, drop-casting Pt-disk electrodes covered by the polymers were used as the working electrode with Ag-wire as the reference electrode, platinum as the counter electrode. The sample was prepared by mixing 1.6 mg PTT-1 (PTT-2, PTT-3 or PTT-4), 1.6 mg acetylene black, 0.32 mg PVDF and 1 mL NMP to a 1.5 mL centrifuge tube, then the mixture was sonicated for 30 min until a well-dispersed solution was formed. Finally, 4 μL of the solution was dropped onto the electrode, and dried at room temperature for the CV test. 0.2 mol/L tetrabutylammonium hexafluorophosphate in acetonitrile solution (TBAPF6/CAN) was used as the electrolyte, with a potential window between -2V-2V (vs. pseudo Ag wire reference electrode), and the sweep rate was 100 mV s^{-1}. All measurements were calibrated against an internal standard of ferrocene (Fc), the ionization potential (IP) value of which is -4.8 eV for the Fc/Fc^+ redox system.

The HOMO/LUMO energy level of the pure polymers can be calculated by:

$$HOMO=-e(Eonset+4.8-0.54) \quad (1)$$

$$LUMO = HOMO + E_g \quad (2)$$

E_g was obtained from the UV-vis absorption spectra. The onset oxidation potential (E_{onset}) vs. Ag wire were obtained from CV curves.

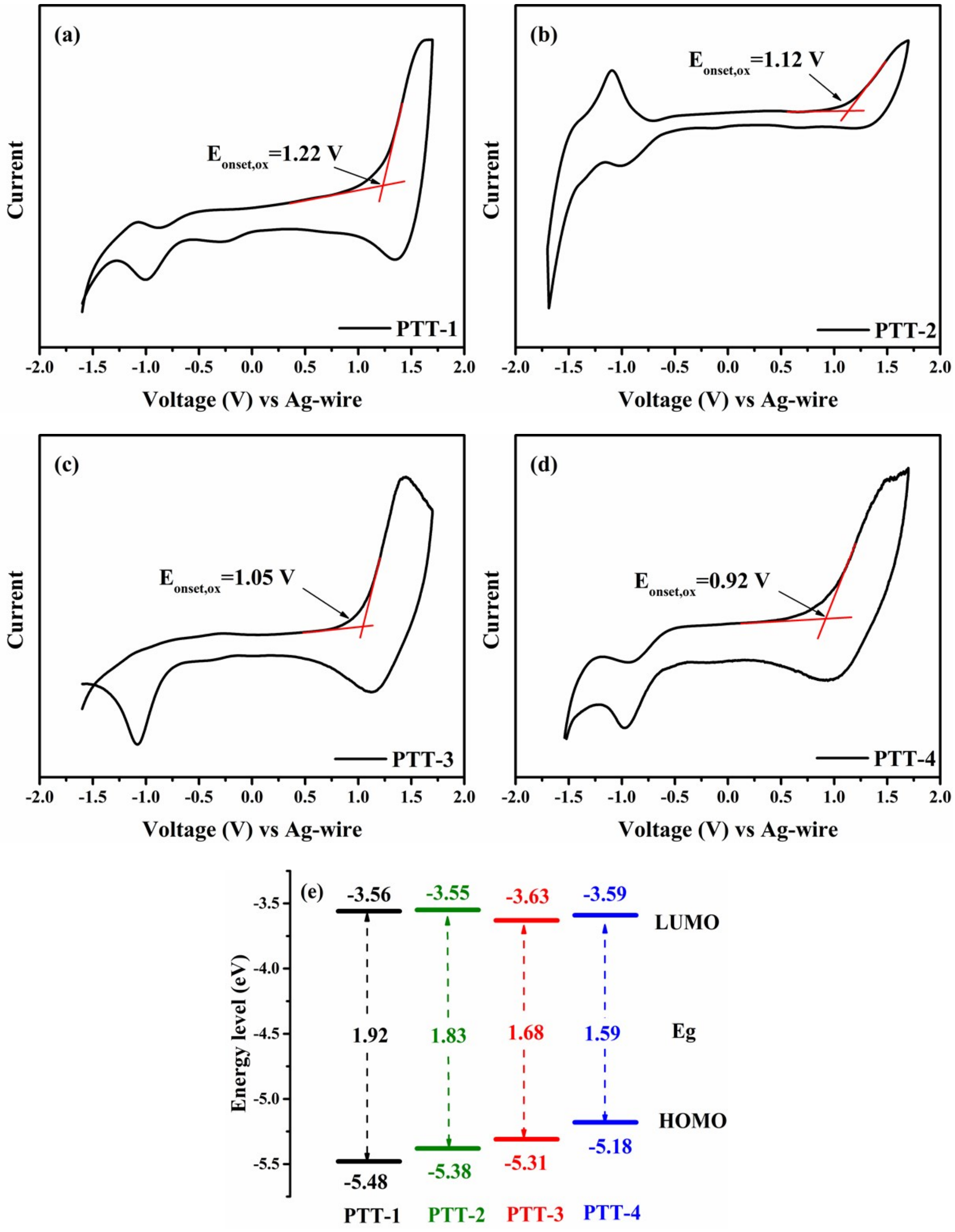

Fig. S9. Cyclic voltammogram of (a) PTT-1, (b) PTT-2, (c) PTT-3 and (d) PTT-4; (e) the orbital

energy level of the polymers.

GDC curves of the composites

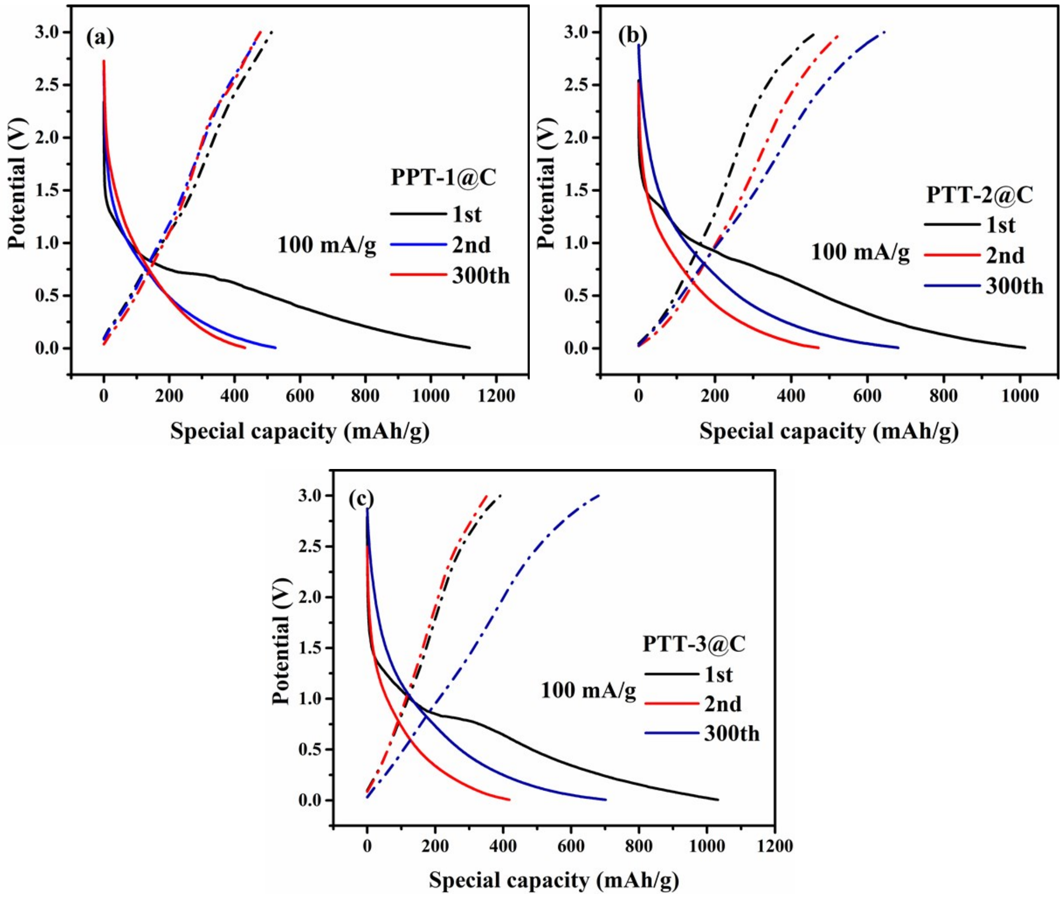

Fig. S10. GDC curves of (a) PTT-1@C, (b) PTT-2@C and (c) PTT-3@C at different cycles, 100 mA/g.

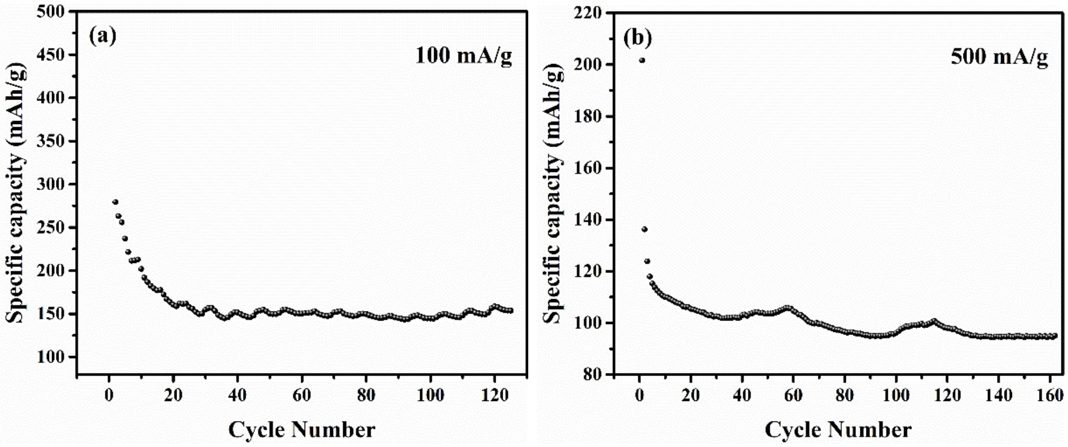

Fig. S11. GDC curves of Vulcan X-72 carbon with different current density 100 mA/g and 500 mA/g.

Synthesis of PTT-5 and PTT-5@C:

300 mg 2,4,6-tris (5-bromothiophen-2-yl)-1,3,5-triazine(TBYT), 392.35 mg 5,5' - bis(triMethylstannyl) - 2,2' - bithiophene (2SnBT) and 60 mg Bis(triphenylphosphine)palladium ($PdCl_2(PPh_3)_2$) catalyst were added to the round-bottom flask, the following produces are the same as the synthesis of PTT-1. For the synthesis of PTT-5@C composites, the mass of the Vulcan X-72 carbon was adjusted to 712 mg, and the mass ratio of the polymer in the composite was 30%.

Synthesis of PTT-6 and PTT-6@C:

300 mg 2,4,6-tris (5-bromothiophen-2-yl)-1,3,5-triazine(TBYT), 459.39 mg 2-(trimethylstannyl)-5-(5-(5-(trimethylstannyl)thiophen-2-yl)thiophen-2-yl)thiophene (2SnTTT) and 60 mg Bis(triphenylphosphine)palladium ($PdCl_2(PPh_3)_2$) were used during the synthesis process, others procedures are the same as PTT-1. For the synthesis of PTT-6@C, the mass of the Vulcan X-72 carbon was 869.2 mg, and the mass ratio of the polymer in the composite was 30%.

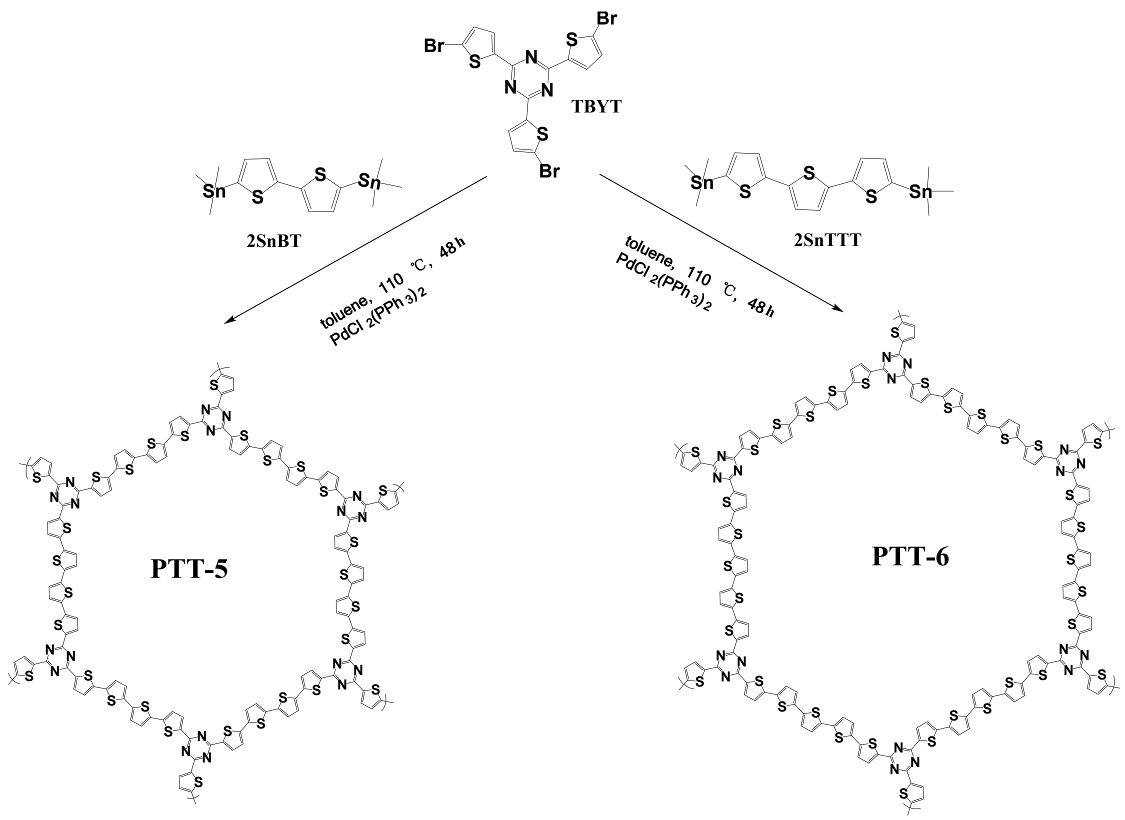

Fig. S12. The synthesis route of PTT-5 and PTT-6.

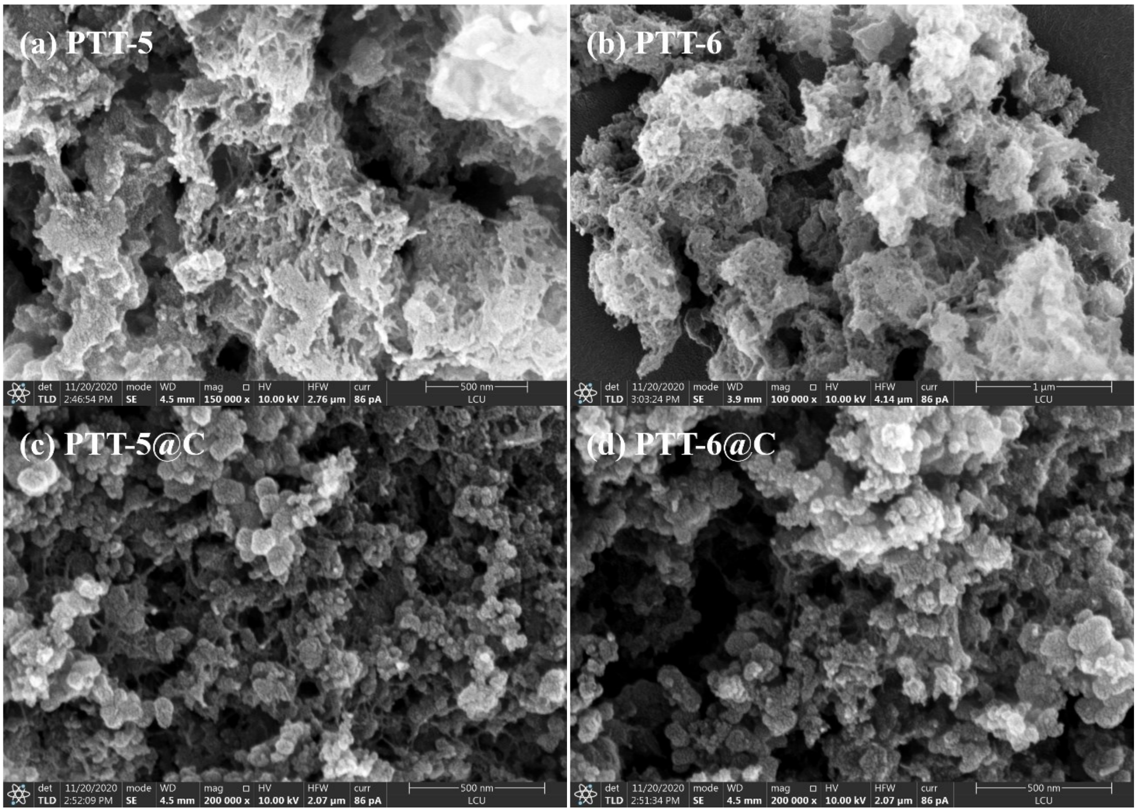

Fig. S13. The SEM images of (a) PTT-5, (b) PTT-5@C, (c) PTT-6 and (d) PTT-6@C

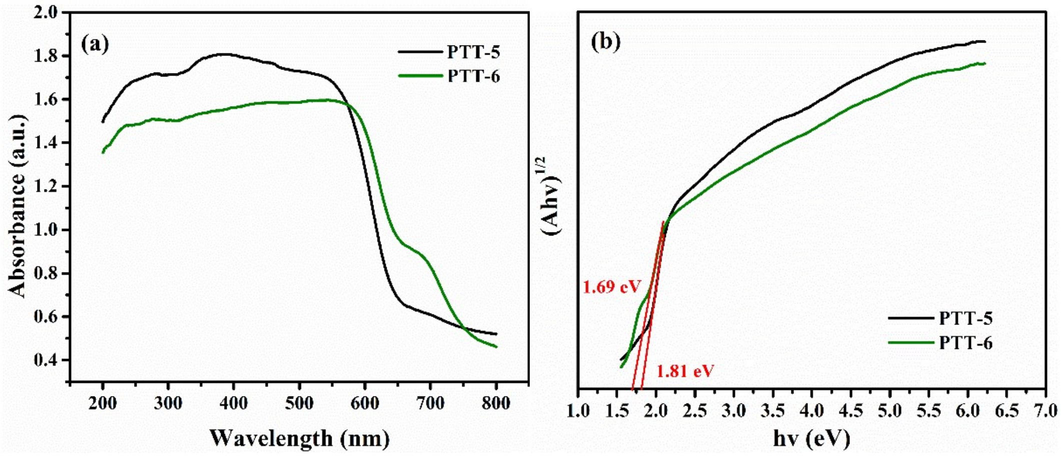

Fig. S14. (a) the UV-Vis spectrum of PTT-5 and PTT-6, (b) the Tauc plot of PTT-5 and PTT-6

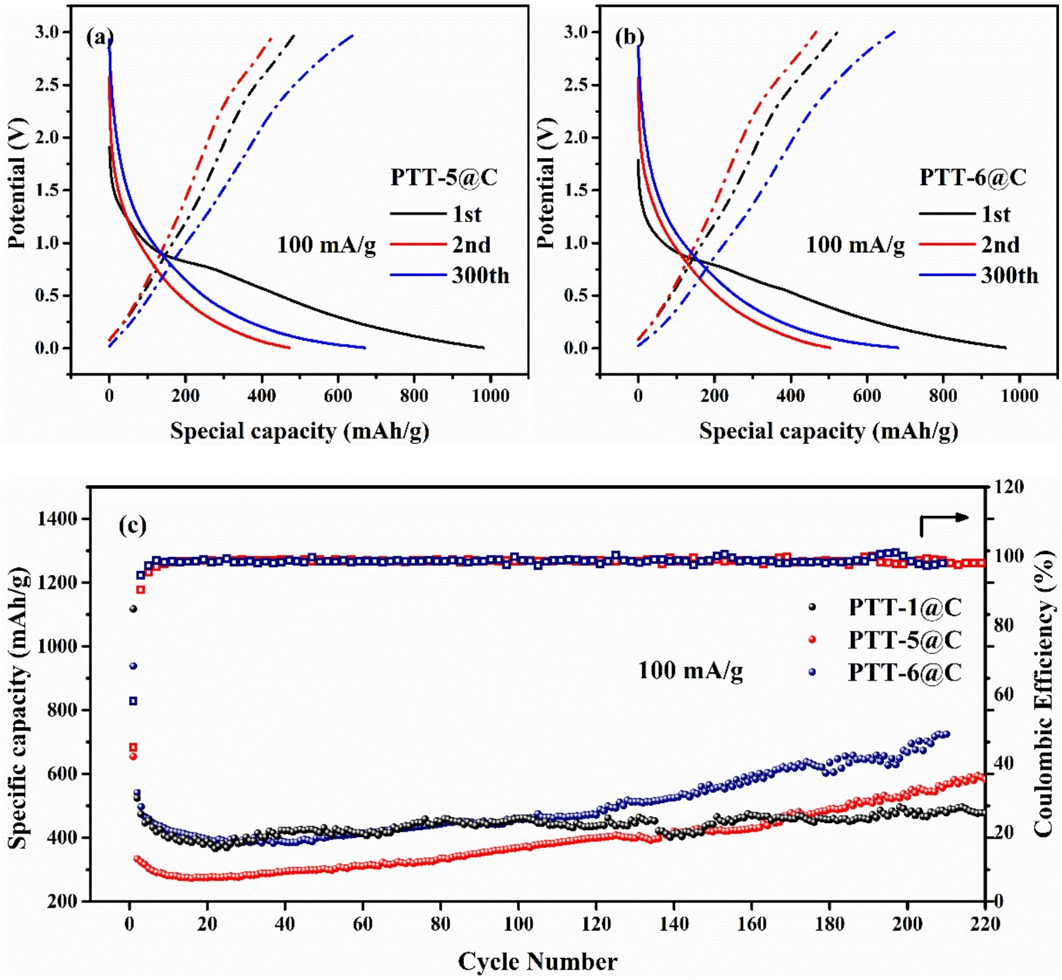

Fig. S15. the GDC curve of (a) PTT-5@C and (b) PTT-6@C at different cycles, 100 mA/g; (c) the cycling performances of the PTT-5@C and PTT-6@C at 100 mA/g
